# A19 INTERLEUKIN-6 AND INTERLEUKIN-22 MEDIATE DISTICT REGULATION OF MITOCHONDRIAL DYNAMICS IN PATHOBIONT INFECTED GUT EPITHELIA

**DOI:** 10.1093/jcag/gwae059.019

**Published:** 2025-02-10

**Authors:** S Navaneetha Krishnan, A Wang, T E Shutt, D McKay

**Affiliations:** Physiology and Pharmacology, University of Calgary, Calgary, AB, Canada; Physiology and Pharmacology, University of Calgary, Calgary, AB, Canada; Biochemistry & Molecular Biology, University of Calgary, Calgary, AB, Canada; Physiology and Pharmacology, University of Calgary, Calgary, AB, Canada

## Abstract

**Background:**

The IBD-associated pathobiont attaching-invading *Escherichia coli* (AIEC) strain LF82 induces mitochondrial fragmentation and depolarization in epithelial cells, resulting in impaired ATP production and reduced barrier function. Interleukin (IL)-6 and IL-22 are omnipresent in the inflamed gut, can directly affect epithelial function and both mobilize STAT-3, the serine727 phosphorylated form of which can translocate to mitochondria. Thus, we posed the question, will IL-6 or IL-22 rescue or exaggerate the mitochondrial damage evoked by exposure to *E. coli*-LF82?

**Aims:**

1. To determine if IL-6 and IL-22 modulate mitochondrial function during *E. coli*-LF82 infection

2. To assess the mechanisms by which IL-6 and IL-22 affect mitochondrial function in model gut epithelia

**Methods:**

Human colon-derived T84 epithelial cells were infected with *E. coli*-LF82 (10^8^ CFU/mL, 4h) ± a 18h pre-treatment and then co-treatment with IL-6 or IL-22 (both 10 ng/mL). Bacterial internalization was evaluated, and mitochondrial network morphology visualized with MitoTracker Red and confocal microscopy. Mitochondrial membrane potential was evaluated using TMRE staining and flow cytometry, and ATP measured by a luminescence assay. STAT3 phosphorylation (S727 and Y705) in whole-cell extracts was determined via western blot in response to IL-6 or IL-22 during *E. coli*-LF82 infection.

**Results:**

IL-6 inhibited *E. coli*-LF82 evoked mitochondrial fragmentation, with significantly more cells showing a fused tubular pattern: mitochondrial membrane potential was restored by ~50% and ATP depletion was less severe (n=5). IL-6 elicited increased pS727-STAT3. In contrast, IL-22 failed to prevent any of the mitochondrial changes caused by *E. coli*-LF82, did not cause increased pS727-STAT3 but bioactivity was confirmed by increased pY705-STAT3. IL-6, but not IL-22, reduced the number of viable intracellular bacteria without affecting bacterial growth and increased markers of autophagy activation.

**Conclusions:**

We have uncovered divergent effects of the STAT3 activators, IL-6 and IL-22, in the control of epithelial mitochondrial dynamics and function when challenged with a bacterial pathobiont. We speculate that IL-6 limits the effects of *E. coli*-LF82 via mitochondrially-directed pS727-STAT3 signaling and upregulation of autophagy. Understanding the precise role of STAT3- activating cytokines signaling in mitochondrial regulation could reveal new target to enhance epithelial barrier function.

**Funding Agencies:**

CIHR

